# Motives and constraints to bike tourism in Greece: “the Go-bike” project

**DOI:** 10.3389/fspor.2024.1475533

**Published:** 2024-11-14

**Authors:** Apostolia Ntovoli, Thomas Karagiorgos, Glykeria Myrovali, Eleni Anoyrkati, Sousana Papadopoulou, Iason Tamiakis, Kostas Alexandris

**Affiliations:** ^1^ Department of Physical Education and Sport Sciences, Frederick University, Limassol, Cyprus; ^2^ Laboratory of Management of Sports Recreation and Tourism, Department of Physical Education and Sport Science, Aristotle University of Thessaloniki, Thessaloniki, Greece; ^3^ Centre for Research and Technology Hellas (CERTH)/Hellenic Institute of Transport (HIT), Thessaloniki, Greece; ^4^ Coventry University Services, Coventry University, Coventry, United Kingdom; ^5^ Department of Nutritional Science and Dietetics, International Hellenic University (IHU), Thessaloniki, Greece; ^6^ Tero Consulting, Thessaloniki, Greece

**Keywords:** bike tourism, barriers, motivation, involvement, technology, mobile phone application, sustainability, tourism participation

## Abstract

**Introduction:**

Bike tourism is one of the fast-developing alternative forms of tourism since it can satisfy the main pillars of sustainability (economic, social, and environmental). The current study is part of a larger funded project (GoBike) and aims to profile bike tourists in Greece, examine the motives and constraints to tourism participation, and show the value of using technology as a means of promoting bike tourism.

**Methods:**

The data was collected through a quantitative study, with one hundred and five individuals who had experience with bike tourism activities, with the use of an online questionnaire. Items were used to measure socio-demographics, motives, constraints, involvement, and attitudes toward a smartphone application.

**Results:**

The results indicated that “Nature”, “Health”, “Bike eco-friendly place” and “Interesting places” were the most important motives. On the other hand, the lack of “Guides”, “Appropriate Routes” “Bike tourism Programs” and “Limited Information” were reported as the most important barriers. The bikers reported that technology can facilitate their decision to do bike tourism activities.

**Discussion:**

A smartphone application should include several attributes the most important of which are the “Elevation difference”, the “warnings of obstacles/risks”, “the level of difficulty”, “the bike distance” and the “condition of the routes”.

## 1 Introduction

The global cycle tourism market size was estimated to be at 345.1 million dollars in 2020 and it is expected to increase to 1,291.3 million dollars by 2030, with a registered CAGR of 14.78% from 2021 to 2030 (1). The revenue in the cycle market is projected to reach 35.52 bn dollars in 2024 and is expected to show an annual growth rate (CAGR 2024–2028) of 0.94%, resulting in a projected market volume of 36.88 bn dollars by 2028 (2). Overall, around 143 million bicycles were sold worldwide in 2022. The largest share of bicycle sales was in Asia, in which China is leading with around 43 million units sold in 2022. The region with the second highest bicycle sales in 2022 was Europe with around 24 million bikes sold (2).

Cycling tourism is a form of alternative tourism that can help the economic development of local communities and attract a large number of tourists throughout the year, as it is not strongly characterized by seasonality (3). It is linked to sustainable development (4). In the context of sustainable tourism, an established trend in developed countries, cycling tourism offers tourists the opportunity to explore and discover little-known areas that are often located in mountains or areas with different characteristics (4, 5). The rising preferences of tourists to get involved in active recreation and adventure sports are the major drivers of the cycle of tourists (6). Considering the increased energy crisis, environmental pollution, and the phenomenon of global warming, biking has been promoted as one of the strategies to reduce all of the above (6, 7). Using bikes as an alternative transportation mode offers several benefits, including but not limited to zero emissions, reduced energy consumption, and enhancements to health and fitness (8, 9). The increased popularity of cycling tourism is also justified by its proven benefits on physical and psychological health (6). The promotion of bicycle tourism presents therefore an opportunity to enhance the tourism sector, mitigate carbon emissions, conserve energy resources, and foster the generation of additional employment prospects (10). For a detailed review of the literature, see the paper of Ciascai et al. (6).

Due to its economic potential and sustainable practices (11), there is a growing focus of the government on the development of cycle tourism resulting in the development of the cycling infrastructure, which fuels the growth of cycle tourism (1, 12). According to the Institute for Transportation & Development Policy (ITDP) cycle generates annual revenues of over EUR 44 billion, an amount that is greater than the revenues of the European cruise-ship industry (13). In the already existing growing trend, the pandemic COVID—19 revealed an additional emerging demand for cycling and offered the opportunity for many countries to redefine the tourism product so that cycling tourism could be strengthened (14). Due to COVID-19, investments of €1 billion have been made in Europe to build cycling infrastructure, creating at least 600 miles (1000 km) of bike lanes (15). Supporters in the effort to develop cycling are not only from traditional countries (Denmark, Holland, etc.) but also countries with insufficient infrastructure that have nevertheless identified the prospects of cycling as a means of transport and as a form of tourism (15, 16). Ciascai et al. (6) reported that cycle tourism has a direct impact on the cycling industry; approximately 3.7 million new bikes were sold (GBP1.62 billion) in 2010 at 2,500 cycle shops across the UK. A study commissioned by the European Parliament (2012) and carried out by the European Cyclists Federation (ECF) (17) estimates that when the EuroVelo network is completed it will generate direct revenues of EUR 7 billion each year, making it possible to service 60 million cycling trips (18). In 2014, the cycling sector was estimated to have around 650,000 full-time jobs (EU-27, excluding Croatia), of which 524,052 jobs were attributed to cycling tourism.

As previously noted, there is an increasing trend for using cycling as a recreational, leisure, and sporting activity concerning sport tourism (19). Research also in this area has been significant in the last 20 years. In a detailed review of the literature, Ciascai et al. (6), identified 680 articles published using as keywords bicycle, tourism, and cycling in 2020–2021. This number shows the growing interest in this area. It is worth noting that 424 of them were published in open-access full-text journals, which shows the increased interest of publishers/authors in open-access journals. In their systematic bibliographic review, these authors found that several terms were linked with bike tourism, such as green tourism, eco-tourism, sport tourism, slow tourism, etc. This shows that bike tourism is part of the sustainable tourism research area.

In Greece, where tourism constitutes the largest economic activity (20–22), accounting for 18%–20% of the national GDP (23), a substantial number of tourists exhibit recently positive attitudes towards alternative and active tourism (24, 25). It is well known that the mass tourism model has traditionally been the tourism development model in the country (26), especially in the Aegean and Ionian islands (27). Nevertheless, considering the social, economic, and environmental perspective of alternative tourism and the negative effects of overtourism, the promotion of small-scale and alternative tourism has been promoted recently (26, 28). Within this framework, niche tourism, characterized by tailored activities aligning with individual visitor preferences, has been developed, aiming to rejuvenate the tourism product and mitigate the seasonality predicament inherent in mass tourism in Greece (29). Cycle tourism is one of the alternative forms of tourism that can satisfy all the characteristics of sustainable tourism (30). Although cycling represents a niche market with considerable potential (31), and Greece can target it, should be emphasized that research is still limited.

The current paper reports the results of the “GoBike” funded project, which aimed to propose good practices for the promotion of bike tourism in Greece. The outcome of this project was to develop and test a mobile phone application, which will not only include navigation attributes but will be an integrated promotional tool for bike tourism providers. This paper reports the profile of bike tourists in Greece, their motives and constraints to participating in bike tourism activities, and their perceptions about the most important figures that such an application should include. There is no published research so far to examine bike tourists’ behavioral and attitudinal (involvement) profile as well as their motives and constraints to participation, in the context of using technology to promote bike tourism.

## 2 Theoretical background

### 2.1 Bike tourism definitions

The multifaceted nature of cycling tourism creates challenges in formulating a universally acknowledged definition. Drawing upon Standeven and Knop's (32), work, sports tourism is elucidated as “sport-based travel away from the home environment for a limited time, where the sport is characterized by unique rule sets, competition related to physical prowess and play” [(33), p. 5]. Within this framework, cycling tourism, a subcategory of sports tourism, was defined by Lamont (19) as “trips away from one's home region with active or passive participation in cycling as the primary purpose” (p. 21). This definition includes race spectators, as well as active participants in both competitive and non-competitive cycling events (34, 35). Simonsen et al. (4) defined a cycling tourist as a person who, during his/her holiday, uses a bicycle as a means of transportation and for whom cycling is an important part of that holiday.

#### 2.1.1 Mountain biking tourism

The mountain biking domain, encompassing its sporting and recreational dimensions, has experienced rapid expansion over the past two decades (12). Mountain biking, originating in the 1980s, has undergone significant development, transforming into a global sport and recreational pursuit (36). According to Siderelis et al. (37), mountain biking was defined as off-road cycling requiring specialized equipment to navigate the remote, rough, and narrow trails that traverse through forests, deserts, mountains, and/or meadows.

Mountain biking is closely linked with tourism if it includes overnight stays (38). Moularde and Weaver (38) defined mountain bike tourism as trips at least 24 h away from a person's home environment for which active participation in mountain biking for recreational purposes is the primary motivation and determining factor in destination choice’ (p. 3). There have been recently several initiatives directed toward fostering mountain bike tourism, primarily through the establishment and promotion of mountain bike trails and parks (12). In the context of specialized travel motivated by mountain biking, the appeal of a destination is an important element (38). Strategies related to mountain bike tourism promotion predominantly center around enticing visitors through the organization of events and the promotion of general visitation to explore diverse trail networks and/or specialized mountain bike parks (38, 39). Still, the main bikers’ motives for participating in biking tourism have not been fully explored in relation to their involvement levels.

#### 2.1.2 Exercise motivation

The nature and strength of motivation have been proposed as the most important facilitators of leisure participation. Bandura (40) conceptualizes motivation as a dynamic process connected to the impetus for action and its subsequent execution. Motivations, perceived as dynamic forces, typically become apparent in response to unfulfilled needs, instigating subsequent actions (41–43). In the realm of tourism research, numerous investigations have delved into the varied motivations underpinning travel (44). The study of these motivations is imperative for marketing professionals, given their influential role in shaping behavior (45). A holistic understanding of participation motivations can facilitate effective market segmentation and the development of targeted marketing campaigns (46, 47).

Initially, scholarly investigations posited the existence of two primary forms of motivation: intrinsic and extrinsic. Intrinsic motivation involves engaging in an activity for its inherent satisfaction and pleasure, as outlined by Deci (48). Individuals intrinsically motivated perform the behavior voluntarily, devoid of external inducements (49). Moving from intrinsic motivation to extrinsic motivation and finally to amotivation, individuals are considered amotivated when there is a noticeable absence of motivation, and the behavior lacks intrinsic or extrinsic rationale (49). In such instances, individuals participate without purpose or expectations, potentially leading to a dropping out of the activity.

This theoretical framework has found extensive application in various leisure-related behaviors (50). The literature on sport and exercise motivation is extensive; it is not the objective of the paper to do a detailed review of this literature. A systematic review of the exercise motivation literature can be found in the studies of Standage and Ryan (51) and Manninen et al. (52). In contrast, extrinsic motivation pertains to behaviors pursued as a means to an end, lacking inherent enjoyment or satisfaction (48).

Expanding on the concept, Deci and Ryan (49) developed the Self-determination Theory, according to which an individual's motivation runs along a continuum, as the individual moves from the stages of amotivation, to extrinsic motivation (control orientation) and finally to intrinsic motivation (autonomy orientation) (49, 53). They propose a continuum of motivation types, ranging from high to low levels of self-determination. Research has shown that motivation is an important factor in predicting exercise commitment and involvement (54). Specifically, the intrinsic motives are the ones that increase exercise loyalty and commitment, while the extrinsic motives do not strongly predict exercise commitment. Individuals who are driven by extrinsic motives might drop out. However, research has also shown that some external motives can be internalized with the time of individuals and can contribute to exercise loyalty when individuals experience the benefits of exercise. For example, exercise “to lose weight” is an external motive, but when an individual feels the benefits of losing weight, it can be internalized (53). The literature on sport and exercise motivation is extensive; it is not the objective of the paper to do a detailed review of this literature. A systematic review of the exercise motivation literature can be found in the studies of Standage and Ryan (51) and Manninen et al. (52). There have been very few studies that examined motives for cycling with the use of self-determination theory (55, 56) but no research in the context of bike tourism.

#### 2.1.3 Leisure constraints

Jackson (57) defined constraints as: “factors that are assumed by researchers and perceived or experienced by individuals to limit the formation of leisure preferences and to inhibit or prohibit participation in leisure activities” (p. 279). Crawford and Godbey (58) classified constraints into structural, interpersonal, and intrapersonal. Structural constraints include external to individual factors such as lack of time, lack of resources, and facility/services problems. Interpersonal constraints relate to the lack of a partner to participate in sport and exercise together. In contrast, intrapersonal constraints are internal and include perceptions related to low self-esteem, limited abilities, and societal values (59, 60). These three categories of constraints were placed within a hierarchical model of leisure decision-making by Crawford et al. (61), based on the way that they influence leisure preferences and actual participation. Intrapersonal constraints are the most powerful in blocking exercise participation, structural constraints are the least powerful in blocking exercise; they rather modify participation. Finally, interpersonal constraints can both block and/or modify participation (61).

Research on leisure constraints has been extended over the last twenty years. A detailed review of the literature can be found in the paper of Funk et al. (62). There have been, however, very limited attempts so far to examine constraints to bike tourism participation, with reference to the hierarchical model of leisure constraints.

#### 2.1.4 Leisure involvement

In the current study, we used the construction of leisure involvement (involvement with bike tourism activities) to examine the influence of motives and constraints on the development of bike tourism involvement. Research has shown that increased involvement is associated with a higher intention to repeat participation (63).

Involvement is described as “a person's perceived recognized past definitions of the object based on inherent needs, values, and interests” [(64), p. 342]. Contemporary research underscores involvement as a critical factor in decision-making for leisure activities, as it correlates with numerous positive behavioral and attitudinal outcomes (63). Studies indicate that involvement is linked to an increased willingness to search for relevant products and services, receptivity to marketing information (65), heightened awareness of a product or brand, loyalty to specific leisure providers or programs (35, 66), and emotional attachment to an activity or setting (67). The commonly utilized dimensions for this purpose are Attraction, Centrality, and Self-expression, as identified in studies by Kyle et al. (67) and other recent research in various leisure contexts (59). Attraction reflects the perceived significance of an activity, and the pleasure derived from participating in it (65). Centrality pertains to the extent to which an activity holds a central role in an individual's life and the social connections related to the activity (e.g., friends, and significant others) (68). Lastly, self-expression gauges the extent to which an activity allows individuals to identify with its values and express their identity through participation (68). Involvement is a psychological variable frequently used to segment leisure participants. Tkaczynski et al. (44) reviewed 119 segmentation studies in tourism, revealing diverse bases and variables used for segmentation. Motivations for travel, influencing travel decisions, and destination choice, have been a common basis for segmentation in numerous studies (69).

## 3 Mobile phone applications for cycle tourism

Using technology is a critical factor today in modern marketing and the delivery of sport tourism services (70). The perceived utilization of smart applications to influence consumer behavior has been identified as a significant factor across diverse contexts (71, 72). Smart applications, herein referred to as mobile applications or apps, represent a pervasive tool used to influence consumer behavior, leveraging their widespread adoption in personal computing and mobile devices for information access and dissemination (73, 74). These apps have progressively assumed a pivotal role in shaping consumer experiences (75, 76), where, for instance, tourists’ inclination towards smartphone usage positively correlates with their inclination to engage in local tourism activities (73). Moreover, consumer innovativeness, exemplified by the utilization of smart apps, significantly impacts travel customer engagement (75), while smart tourism applications directly influence the perception of smart destination image (76). By facilitating map display, scale manipulation, and movement tracking, mobile applications wield significant influence over cycling behavior through goal setting, self-monitoring, and feedback provision (77).

Consequently, smart applications extend beyond the mere provision of information regarding specific attractions or locations, or the recommendation of destinations and itineraries, including cycling tours (78). Even though the constraints factors for participation in mountain biking tourism have been studied (79), to a limited extent, a larger gap is identified in the literature regarding whether a smart mobile phone application could offer to reduce these barriers and increase bikers’ motivations.

## 4 Research objectives

Following the above discussion, the research objectives were set as follows:

• To profile bike tourists based on socio-demographic characteristics
• To investigate the motives for bike tourism and the constraints to participate in bike tourism activities.
• To profile the bike tourists according to their involvement levels
• To test how motives differ in terms of biking involvement levels
• To report the characteristics/attributes of a smartphone application for tourism bikers.

Based on the above objectives the following research questions were set:

• What is the socio-demographic profile of Greek bike tourists?
• What are the motives for participation and constraints that prohibit participation?
• How can the concept of involvement be used to cluster tourism bikers?
• What are the motives that can differentiate between high and low-involved tourism bikers?
• What are the most important characteristics/attributes of a smartphone application for tourism bikers?

## 5 Methodology

The data was collected with a quantitative survey in 2022 of Greek individuals who had some experience in mountain bike tourism activities. An online questionnaire was developed. Since there was no “formal” blog or association to represent mountain bike tourists in Greece, the online questionnaire was distributed through the social media (Facebook and Twitter) of the partners of the project, and some informal blogs of mountain bike tourists. These blogs were identified after consultation with professional private and public agencies that offer mountain bike tourist activities in Greece. Bike tourists were invited to fill out the questionnaire which was sent in a specific link that was created. Two research assistants had access to the questionnaires answered and the database. The data were then inserted into SPSS to do the statistical analysis.

It has to be noted that it was not possible to find a list of blogs and platforms that related to mountain bike tourism in order to choose some of them randomly. Following the above method, we achieved a sample of one hundred and five individuals (*N* = 105) who had experience in bike tourism activities. The non-probability sample is one of the limitations of the study. Since this is a non-random sample, it cannot be considered representative of the study population. Results therefore cannot be generalized with confidence. They, however, show some trends.

A consent form was first completed before they filled out the questionnaire. A screening question was also included in order to have in the sample only those who had participated at least once during the previous year in bike tourism-related activities. A short explanation of what is considered bike tourism activities was also given as an opening paragraph.

The involvement scale was measured with 6 items, following Kyle et al.'s (80) questionnaire (attraction, centrality, and self-expression), the motives for mountain bike tourism were selected from Manfredo's (81) recreation experience scale, while constraints were measured with an adjusted version of Alexandris & Carroll's (82) scale. Finally, bikers’ views towards the phone application attributes were measured with a twenty-item questionnaire which was developed for the purposes of the study.

### 5.1 Data analysis

Descriptive statistics were used to profile mountain bike tourists and present the results in motivation, constraint, involvement, and smartphone application questionnaires. Both the Kolmogorov-Smirnov and Shapiro-Wilk tests were used to test the normality of the data. Cluster analysis, using computed involvement factors, was used to classify participants into segments. Mann-Whitney *U* tests were used to compare high and low-involved individuals in the motives and constraint scores. The Mann-Whitney *U* test was used because of the non-probability sample and because the data did not satisfy the normality condition. The Mann-Whitney *U* test is used to compare differences between two independent groups when the dependent variable is either ordinal or continuous, but not normally distributed (83). In terms of its assumptions, our data are continuous (interval), and variables are independent (categorical, cluster groups).

Finally, effect size was tested with the rank-biserial correlation coefficients, which is the most common method to test effect size in non-parametric tests. It provides an estimate of the strength and direction of the relationship between the independent groups and the outcome variable. The rank-biserial correlation coefficient ranges from −1 to 1, where −1 indicates a perfect negative relationship, 0 signifies no relationship, and 1 denotes a perfect positive relationship. It is calculated based on the *U* statistics from the Mann-Whitney *U* test and specific formulas depending on the test version and sample sizes (84). A typical norm is that correlations more than ±.7 are strong, ±.4 to ±.6 are moderate and ±.1 to ±.3 are weak (85).

## 6 Results

The age of the respondents was coded in nominal categories, as follows: 18–24, 25–34, 35–44, 45–54, 55–64 and >65. Six levels of occupation were included, as follows: university students, private sector, public sector, entrepreneurs, unemployed, and others. The demographic characteristics of the sample are presented in Table 1. Most of the sample was men (68.8%). Regarding the age group, about two-thirds of the sample were between 35 and 44 (32.1%) and 45–54 (30.2%), followed by 25–534 (27.4%). Concerning occupational status, the majority were working as entrepreneurs (35.8%), followed by those who were working in the private sector (34%) and those who were employed in the public sector (20.8%), while the sample was almost balanced among those who were university students (2.8%), unemployed (2.8%) and to those who were employed in “other” occupations (3.8%) (Table 1). Regarding the educational groups, half of the sample was master's degree holders (50%), followed by graduates’ students (34.9%), those who were at vocational (8.5%) and secondary level (6.6%). While concerning marital status the vast majority of the samples were married (55.7%), followed by those who were single (36.8%) and those who were divorced (7.5%).

**Table 1:** Demographic characteristics of the sample.

Gender groups	Age groups		Occupation	
Males 68.8%	18–24	3.8%	University students	2.8%
Females 31.2%	25–34	27.4%	Private sector	34%
	35–44	32.1%	Public sector	20.8%
	45–54	30.2%	Entrepreneur	35.8%
	55–64	6.6%	Unemployed	2.8%
	>65	–	Other	3.8%

### 6.1 Behavioral profile

The results indicated that 41% of the respondents participated less than once a week in tourism bike programs, 34% participated 1–2 times a week, 16% participated 3–5 times a week, and 14% participated almost every day (Table 2).

**Table 2:** Frequency of biking.

Valid	Frequency	Percent	Valid Percent	Cumulative Percent
1–2 times per week	34	32.4	32.4	32.4
3–5 times per week	16	15.2	15.2	47.6
Less than once per week	41	39.0	39.0	86.7
Almost every day	14	13.3	13.3	100.0
Total	105	100.0	100.0	

### 6.2 Descriptive statistics

#### 6.2.1 Motivation

In terms of the motivation scale, the mean scores of all item components were average to high. The highest scores were obtained for “Nature” (mean 6.28) and “Health” (mean 6.11), followed by “Bike eco-friendly” (mean 5.83) and “Interesting places” (mean 5.77). The lowest score, which is also high, was obtained for “Family/friends” (mean 4.95) (Table 3).

**Table 3:** Mean scores and SDs of the motivation items.

Motivation scale	Mean	SD
Nature	6.28	1.32
Health	6.11	1.34
Bike eco-friendly	5.83	1.45
Interesting places	5.77	1.48
Outdoor activities	5.65	1.61
Enjoyment	5.58	1.63
Ability	5.45	1.51
New people	5.22	1.61
Popular bike routes	5.17	1.61
Challenge	5.11	1.66
Family/friends	4.95	1.72
Nature	6.28	1.32
Health	6.11	1.34
Bike eco-friendly	5.83	1.45
Interesting places	5.77	1.48
Outdoor activities	5.65	1.61
Enjoyment	5.58	1.63
Ability	5.45	1.51
New people	5.22	1.61
Popular bike routes	5.17	1.61
Challenge	5.11	1.66
Family/friends	4.95	1.72

#### 6.2.2 Constraints

In terms of the constraints scale, the mean scores of all item components had average scores. The highest scores were obtained for “Quailed Guides” (mean 5.05) and “Appropriate Routes” (mean 4.95), followed by “Bike Tourism Programs” (mean 4.57) and “Limited Information” (mean 4.57). The lowest score was obtained for “Expensive” (mean 3.32) (Table 4).

**Table 4:** Mean scores and SDs of the constraint items.

Constraints scale	Mean	SD
Time	4.15	1.96
Bike tourism programs	4.57	2.00
Safety	3.98	1.82
Appropriate routes	4.95	1.82
Tiring	2.39	1.64
Lack of knowledge	4.12	2.23
Limited information	4.57	2.22
Qualified guides	5.05	1.86
Appropriate routes	4.31	1.88
Partners	3.61	2.14
Expensive	3.32	1.88

#### 6.2.3 Involvement

Regarding the involvement scale the highest scores were reported in “Mountain Tourism Biking (MTB) is very Important to me (Attraction_3)” (mean 5.69), “MTB is one of the most Satisfying things that I do (Attraction_1)” (mean 5.50), and “MTB is one of the most Enjoyable things that I do (Attraction_2)” (mean 5.39) followed by “Participation in MTB says a lot about whom I am (Self-Expression_2)” (mean 4.21), “My life is organized around MTB (Centrality_1)” (mean 4.08) and “I can really be myself when I do MTB (Self-Expression_1)” (mean 4.03) (Table 5).

**Table 5:** Mean scores and SDs of the involvement items.

Involvement scale	Mean	SD
Mountain Tourism Biking (MTB) is one of the most Satisfying things that I do (Attraction_1)	5.50	1.70
MTB is one of the most Enjoyable things that I do (Attraction_2	5.39	1.77
MTB is very important to me (Attraction_3)	5.69	1.64
My life is organized around MTB (Centrality_1)	4.08	2.11
MTB occupies a central role in my life (Centrality_2)	3.54	2.09
To change my preference for another tourism activity would require major rethinking (Centrality_3)	2.84	2.03
I can really be myself when I do MTB (Self-Expression_1)	4.03	2.02
Participation in MTB says a lot about whom I am (Self-Expression_2)	4.21	1.98
When I participate in MTB others see me the way I want them to see me (Self-Expression_3)	4.01	2.22

Both Kolmogorov-Smirnov and the Shapiro-Wilk tests were used to test the normality of the data (involvement, constraint, and motives variables). The sig. value of the test was lower than.05 in all the items (.001 in all of them), showing that the data significantly deviated from normal distribution.

### 6.3 Cluster analysis

A cluster analysis, using the computed involvement factors, was employed to classify participants into segments. The alpha scores of the involvement factors were all high (>.91, Table 6). The Ward method using K-means clustering was used. The analysis indicated that a two-group solution was the most meaningful. The two segments were defined as Low Involvement (*N* = 44) and High Involvement (*N* = 61). A Mann-Whitney *U* test showed that they differed significantly in all three involvement dimensions (Table 6). The rank-biserial correlation coefficients, as an indication of effect size range from.80 to.84, showing significant strong correlations (last column).

**Table 6:** Clusters of bikers in terms of involvement levels.

	Internal consistency	K-means cluster analysis			
		Cluster 1: Mean rank/sum of rank	Cluster 2: Mean rank/sum of rank		
	Cronbach's alpha	High involved (*N* = 61)	Low involved (*N* = 44)	*Z* scores and probability level	Spearman's correlations
Involvement—attraction	.97	72.8 4,446.5	25.4 1,118.5	128.5 .001	.80***
Involvement—centrality	.91	73.9 4,508.5	24.1 1,056.5	65.5 .001	.81***
Involvement self-expression	.94	74.4 4,544.0	23.2 1,021.0	31.0 .001	.84***

*** .001 level.

#### 6.3.1 Cluster groups and motivation

A Mann-Whitney *U* test was performed to compare motivation scores between high and low-involved groups’ scores. A Mann-Whitney *U* test is used to compare the differences between two independent samples when the sample distributions are not normally distributed. The results revealed statistically significant differences almost in all items from the motivation scale. With the highest scores in the Popular Hike Routes (z = 5.2, *p* < .001), Enjoyment (z = 4.3, *p* < .001) and Ability (z = 4.2, *p* < .001) scales (Table 7). The rank-biserial correlation coefficients are also presented in Table 7 (last column). As seen, they range from.34 to.51, showing significant and moderate correlations.

**Table 7:** Comparison between high and low involved bikers in motivation scale **(**Mann-Whitney test) and Spearman's correlations.

		High involved, *N* = 61	Low involved, *N* = 44	Z scores & probability level	Spearman's correlations
Challenge	Mean rank	61.7	40.8	3.5***	.34***
	Sum of rank	3,768.5	1,796.5		
Ability	Mean rank	63.3	38.6	4.2***	.41***
	Sum of rank	3,863.5	1,701.5		
Family/friends	Mean rank	62.3	40.0	3.7***	.36***
	Sum of rank	3,802.0	1,763.0		
New people	Mean rank	63.4	38.5	4.2***	.41***
	Sum of rank	3,870.0	1,695.0		
Interesting places	Mean rank	63.1	38.8	4.2***	.42***
	Sum of rank	3,854.5	1,710.5		
Popular bike routes	Mean rank	65.8	35.1	5.2***	.51***
	Sum of rank	4,017.0	1,548.0		
Outdoor activities	Mean rank	62.2	40.2	3.8***	.37***
	Sum of rank	3,794.0	1,771.0		
	Sum of rank	3,878.5	1,686.5		
Bike eco-friendly	Mean rank	59.6	43.7	2.8**	.27**
	Sum of rank	3,640.0	1,925.0		
Health	Mean rank	62.9	39.2	4.4***	.43***
	Sum of rank	3,839.5	1,725.5		
Nature	Mean rank	61.9	40.5	4.3***	.42***
	Sum of rank	3,779.5	1,785.5		

*p < .05.

** *p* < .01.

*** *p* < .001.

#### 6.3.2 Cluster groups and constraints

A Mann-Whitney *U* test was performed to compare motivation scores between high and low-involved groups’ scores. The results revealed statistically significant differences in almost all the appropriate routes (*z* = 2.9, *p* < .001), bike tourism programs (z = 2.8, *p* < .001), and tiring (z = 2.5, *p* < .001) items (Table 8). The rank-biserial correlation coefficients are also presented in Table 8 (last column). As seen, they range from -.03 (n.s.) to -.28 (negative significant, with non-significant and low correlations).

**Table 8:** Comparison between high and low involved bikers in constraints scale **(**Mann-Whitney test) and Spearman's correlations.

		High involved, *N* = 61	Low involved, *N* = 44	Z scores & probability level	Spearman's correlations
Time	Mean rank	55.4	49.5	−.98, n.s.	.09
	Sum of rank	3,383.5	2,181.5		
Bike tourism programs	Mean rank	60.1	43.2	−2.8**	−.27**
	Sum of rank	3,660.5	1,904.5		
Safety	Mean rank	57.3	46.9	−1.7, n.s.	−.18
	Sum of rank	3,500.0	2,065.0		
Appropriate routes	Mean rank	60.3	42.8	−2.9, **	−.28**
	Sum of rank	3,678.0	1,887.0		
Tiring	Mean rank	61.4	46.9	−2.5*	−.24*
	Sum of rank	2,863.5	2,701.5		
Lack of knowledge	Mean rank	53.8	51.8	−.34, n.s.	−.03
	Sum of rank	3,286.0	2,279.0		
Limited information	Mean rank	55.7	49.2	−1.0, n.s.	−.15
	Sum of rank	3,399.0	2,166.0		
	Sum of rank	3,545.5	2,019.5		
Appropriate routes	Mean rank	56.8	47.7	−1.5, n.s.	−.15
	Sum of rank	3,466.0	2,099.0		
Partners	Mean rank	55.0	50.1	−.84, n.s.	−.82
	Sum of rank	3,360.5	2,204.5		
Expensive	Mean rank	60.2	42.9	−2.9**	−.29**
	Sum of rank	3,673.5	1,891.5		

* *p* < .05.

** *p* < .01.

***p < .001.

### 6.4 Characteristics/attributes of a good smartphone application for mountain bikers

As shown in Table 9, elevation difference (89%), warnings of obstacles/risks (89%), level of difficulty (88%), bike distance (88%), and condition of the routes (88%) were reported to be the most important features of the application. However, except for the ads, all the features included in the questionnaire were reported by bikers to be important (Table 9).

**Table 9:** Characteristics/attributes of a good smartphone application for mountain bikers.

Attributes	Percent
Elevation difference	89.20%
Warnings of obstacles/risks	89.20%
Level of difficulty	88.10%
Bike distance	88.10%
Condition of the routes	88.10%
Type of route	87%
Water availability	87%
Suggested routes	84.90%
GPS	84.90%
Evaluation based on the experience	84.90%
Elevation gradient	83.80%
Databases (OpenStreetMap etc.)	83.80%
Personalized planning of routes	80.60%
Interesting points/pictures	78.40%
Biking statistics	77.40%
Supporting services	76.30%
Blogs: advise for local visits	70.90%
Pictures	70.90%
Speed	68.80%
Promotional activities	66.60%
Calories	58%
Communication with other bikers	55.90%
Ads	37.60%

## 7 Discussion

### 7.1 The profile of bike tourists

Although the sample of the study was not randomly taken, and results should be generalized with caution, some trends can be pointed out. First, the education variable showed that most tourist bikers are highly educated (at graduate and postgraduate levels). Furthermore, they belong to the 35–55 age group. These results confirm previous studies which proposed that biking tourism is a niche market, which however can be considered as upscale tourism, an attractive element for quality tourism development (86). The bikers were also shown to have families, which once again shows the attractiveness of this market. As expected, they use biking as a frequent leisure activity in their everyday life.

### 7.2 Motives for participation

The descriptive statistics of the motives scale revealed that the needs “to enjoy nature”, “to stay healthy” and “to spend time in ecofriendly environments” had the highest mean scores, which show that bikers are environmentally sensitive and alternative tourists (87). It is therefore expected that bike tourism should be developed following the principles of sustainability: social, economic, and environmental (88). They also show that for bikes caring about their health is one of their life values and a life philosophy. Discussing these results within the framework of the self-determination theory, it can be argued that these motives belong to the intrinsic dimension (48). As previously discussed, intrinsic motivation involves engaging in an activity for its pleasure and satisfaction (48). Intrinsically motivated behavior is performed voluntarily. These self-determined motives are not linked with external rewards. Intrinsic motivates are the most likely to lead to repeated behavior, loyalty, and commitment to an activity (54). Subsequently, in the context of mountain biking, they are very likely to lead to positive behavioral outcomes, such as loyal tourism bikers. It must be noted that, as previously discussed, previous studies in exercise motivation did not use items related to enjoyment of nature and eco-friendly environments. However, motives of this nature fit with the definition of self-determined behavior (48). Subsequently, the current study proposes some new items that can be included within the scales that are used to measure leisure motivation, in relation to activities that take place in nature.

It should also be noted that the motive of “visiting interesting places” had also a high mean score, which shows the importance of developing biking routes that connect historical, natural, and even other tourist attractions. Still, this item relates to intrinsically motivated behavior. Recent studies have linked, for example, cycling tourism with wine tourism (e.g., visiting wineries) (84), city tourism (e.g., cycling routes to visit attractions within the city), and outdoor activities (e.g., birdwatching). On discussing the above motives with reference to the self-determination theory it is clear that the majority of the motives belong to the intrinsic dimension, which was expected considering that tourism is a freely chosen leisure activity (89).

In terms of the involvement profile, the cluster analysis revealed two involvement groups almost equal in size, the high and the low involved ones. While it is promising that there is a group of bikers who seem to be highly involved in biking tourism, another group also seems to be less involved. These individuals have tried bike tourism in the past, but, likely, they will not try again in the future (or they will do it with low frequency). Subsequently, it is important to further examine how motives differ between the two groups.

The results of the Mann-Whitney *U* test revealed statistically significant differences between the two groups in all the motivational items, which shows the value of motivation research for understanding tourism behavior. The biserial correlations confirmed these differences, showing significant and moderate effect sizes. Once again, all these are intrinsic motives, which supports the motivation literature, in which it has been reported that intrinsic motivation leads to the exercise of commitment and loyalty (90–92). These results suggest that when developing strategies to promote bike tourism providers should consider the motives for participation and should create situations to foster them. Bikers should have the opportunity to enjoy nature in eco-friendly environments, perceive the health benefits of cycling, enjoy themselves but also visit interesting places, and improve their biking-related skills. It must be noted that mountain biking is one of those sports that require continuous improvement. Even the most experienced riders might have something that they need to improve. Examples can be balanced, jumping the bike off the ground without the help of any type of ramp or jump, sprinting, trail braking, riding on sand or snow or ice, riding while exhausted, etc. (https://simplemtb.com/mountain-biking-skills/. Improving riding skills keeps mountain biking fresh and exciting. It indicates intrinsically motivated behavior, which can lead to loyalty and commitment to the activity (52, 93–95).

While motivation research is important to understand tourism behavior, the results of our study also showed that several factors limit bikers’ intention to participate in such activities. Most of these factors relate to a lack of appropriate routes, programs, information, lack of information, and appropriate programs. Recent studies have emphasized the value of technology in promoting tourist activities (96) effectively. On discussing this result with reference to the hierarchical model of leisure constraints, we can say that most of these factors can be considered structural constraints, as defined by the hierarchical model of leisure constraints (82). This obviously relates to the context of the study. Biking is an activity that requires investments in routes, and information equipment that will create also a perception of safety. It seems that interpersonal constraints are not very important among bikers, since they do have their own communities and groups. These people are also active bikers in their daily lives. Subsequently, the biking infrastructure is the crucial factor at this stage. Constraints however related to safety belong to intrapersonal constraints, according to the hierarchical model of leisure constraints (82). If these constraints are not resolved, they will block participation. It was previously discussed that intrapersonal constraints are the most powerful ones, in terms of controlling leisure behavior. They are the most likely to block leisure participation and not just modify it (82). So, if they are not removed, bikers will not participate in mountain bike tourism activities, even if they express an intention to do so. The infrastructure (routes) and the tourism bike services/programs are important constraints to participation as well as the physical requirements of the activity. These results support the value of using technology to map biking routes so that prospective users can plan their trips. The results of the Mann-Whitney *U* test also confirmed that structural and intrapersonal constraints are those that can differentiate between the high and the low-involved bikers. The biserial correlations, however, showed low to medium effect sizes. It is common in previous constraint research to find weak correlations between perceived constraints and actual behavior [see (28), 60]. This was explained by the negotiation proposition, according to which individuals tend to report structural constraints, which however had been overcome internally with the use of appropriate negotiation strategies. This is the reason that it has been proposed that intrapersonal constraints are the most powerful in blocking leisure participation.

In the current study, it was revealed that a smartphone application should be tailored to satisfy their specific needs. More specifically, the highest scores were given to the ability of the application to show the elevation difference, the warning of obstacles/risks, the level of difficulty, the bike distance, but also the condition of the routes. As it is seen most of these attributes relate to the risks felt by bikers when biking routes are not well maintained or well-defined, considering that bike tourism is a family activity that should be safe and not very physically challenging. It should be noted that information related to support services, interesting places, and pictures from the route were scored high by bikers, which supports our argument that such applications should be tailor-made and should be used as promotional tools from tourism providers.

This study has practical implications for agencies and tourism providers. First of all, it shows that a demand for mountain bike tourism exists and should be addressed. Second, it shows that mountain tourism biking is closely linked with sustainability perceptions and image, which suggests that it can be one of the future alternative tourism activities that can be included within the sustainable tourism pillars. However, several issues need to be addressed and improved, as bike tourists reported in the constraint questions. Biking routes must be provided, promoted with technology, and linked with other forms of special tourism (e.g., agro-tourism, wine tourism, etc.). Agencies and tourism providers should use phone applications like the one provided by the Go Bike project in order to promote the activity in a modern way and help current and future bikers overcome several constraints related to safety, lack of information, difficulties in planning the tours, etc.

In conclusion, this study, which is part of a larger project, indicated that tourism bikers have an attractive socio-demographic profile for tourism purposes. They belong to middle to high socio-economic groups, and since it is an expensive activity; they are ready to spend money to enjoy mountain biking. Furthermore, they are a dynamic group in terms of their age, since they are young to middle-aged individuals. Finally, they see mountain tourism biking as a social and/or family activity, which is desirable for tourism purposes. In terms of their attitudes, they are environmentally sensitive, driven mainly by motives related to enjoying nature, but they also expect to visit interesting places. They have difficulties in finding information related to bike tourism services, routes, and programs. They are also worried about the safety of the bike routes. Finally, tailor-made smartphone applications are welcome and should include a variety of features related to both the information about the routes and the supporting services.

### 7.3 Study limitations and future research

This study is one of the few ones that specifically investigated the motives and constraints of bike tourism. However, several limitations should be addressed. The sample of the study was a convenient one which means that results cannot be generalized with confidence. The size of the sample is a second source of sampling error. Furthermore, only Greek bikers were included in the sample. Future studies should study international tourists’ perceptions since the Greek tourism industry is mainly driven by international tourists. The final goal of this project was to develop a smartphone application that can facilitate bikers’ participation and promotion. The evaluation of this application is not presented in this study, and this is a topic for future research. Finally, tourism bike experience was not investigated in this study, which is a topic for future research to evaluate the service elements of the activity.

## Data Availability

The raw data supporting the conclusions of this article will be made available by the authors, without undue reservation.
